# Early Life Factors and Inter-Country Heterogeneity in BMI Growth Trajectories of European Children: The IDEFICS Study

**DOI:** 10.1371/journal.pone.0149268

**Published:** 2016-02-22

**Authors:** Claudia Börnhorst, Alfonso Siani, Paola Russo, Yannis Kourides, Isabelle Sion, Denés Molnár, Luis A. Moreno, Gerardo Rodríguez, Yoav Ben-Shlomo, Laura Howe, Lauren Lissner, Kirsten Mehlig, Susann Regber, Karin Bammann, Ronja Foraita, Wolfgang Ahrens, Kate Tilling

**Affiliations:** 1 Leibniz Institute for Prevention Research and Epidemiology – BIPS, Bremen, Germany; 2 Unit of Epidemiology and Population Genetics, Institute of Food Sciences, National Research Council, Avellino, Italy; 3 Research and Education Institute of Child Health, Strovolos, Cyprus; 4 Department of Public Health, Ghent University, Ghent, Belgium; 5 Department of Pediatrics, University of Pécs, Pécs, Hungary; 6 GENUD (Growth, Exercise, Nutrition and Development) Research Group, Faculty of Health Sciences, University of Zaragoza, Zaragoza, Spain; 7 IIS Aragón (Aragon Health Research Institute), Zaragoza, Spain; 8 School of Social and Community Medicine, University of Bristol, Bristol, United Kingdom; 9 MRC Integrative Epidemiology Unit at the University of Bristol, Bristol, United Kingdom; 10 Section for Epidemiology and Social Medicine, Department of Public Health and Community Medicine, Institute of Medicine, Sahlgrenska Academy, University of Gothenburg, Gothenburg, Sweden; 11 School of Health and Welfare, Halmstad University, Halmstad, Sweden; 12 Institute for Public Health and Nursing Research (IPP), Faculty for Human and Health Sciences, University Bremen, Bremen, Germany; 13 Faculty of Mathematics and Computer Science, University of Bremen, Bremen, Germany; Karolinska Institutet, SWEDEN

## Abstract

**Background:**

Starting from birth, this explorative study aimed to investigate between-country differences in body mass index (BMI) trajectories and whether early life factors explain these differences.

**Methods:**

The sample included 7,644 children from seven European countries (Belgium, Cyprus, Germany, Hungary, Italy, Spain, Sweden) participating in the multi-centre IDEFICS study. Information on early life factors and in total 53,409 repeated measurements of height and weight from 0 to <12 years of age were collected during the baseline (2007/2008) and follow-up examination (2009/2010) supplemented by records of routine child health visits. Country-specific BMI growth curves were estimated using fractional polynomial mixed effects models. Several covariates focussing on early life factors were added to the models to investigate their role in the between-countries differences.

**Results:**

Large between-country differences were observed with Italian children showing significantly higher mean BMI values at all ages ≥ 3 years compared to the other countries. For instance, at age 11 years mean BMI values in Italian boys and girls were 22.3 [21.9;22.8; 99% confidence interval] and 22.0 [21.5;22.4], respectively, compared to a range of 18.4 [18.1;18.8] to 20.3 [19.8;20.7] in boys and 18.2 [17.8;18.6] to 20.3 [19.8;20.7] in girls in the other countries. After adjustment for early life factors, differences between country-specific BMI curves became smaller. Maternal BMI was the factor being most strongly associated with BMI growth (p<0.01 in all countries) with associations increasing during childhood. Gestational weight gain (GWG) was weakly associated with BMI at birth in all countries. In some countries, positive associations between BMI growth and children not being breastfed, mothers’ smoking during pregnancy and low educational level of parents were found.

**Conclusion:**

Early life factors seem to explain only some of the inter-country variation in growth. Maternal BMI showed the strongest association with children’s BMI growth.

## Introduction

The prevalence of childhood overweight and obesity differs considerably between European countries [1,2]. These differences start early in life, i.e. growth trajectories of height, weight or body mass index (BMI) during infancy and childhood differ markedly between populations [3]. Several factors related to individual growth have been discussed in the literature including early life factors [4–6] like gestational weight gain (GWG) [7] and breast feeding duration [6], genetics [8], parental adiposity and body size [9], environmental, social [10–12], cultural and lifestyle factors such as physical activity and dietary behaviour [13,14]. Childhood growth is increasingly recognised as an important exposure or mediator for later outcomes like obesity and its comorbidities [15]. Although various factors related to overweight and growth have been identified, little is yet known about the factors explaining the observed inter-country heterogeneity. From a public health perspective identification of modifiable factors affecting growth is of great relevance as these would allow prevention of unfavourable development [16]. There is growing evidence that obesity risk becomes manifest already in the first years of life which stresses the importance of early life factors [17–20]. Exposure to early life factors may differ between countries, partially due to differences in country-specific social norms, policies and guidelines [21–23]. For instance, recommendations but also observed durations of breast feeding [24,25], the prevalence of smoking during pregnancy [26–28] and parental overweight/obesity [29] all differ between European countries. In addition, exposures to early life factors may have changed over time due to secular trends. For smoking during pregnancy decreasing trends were reported for some, but not all, developed countries over the last decades but large differences in pregnancy smoking prevalence persist [27]. Recent comparative data on alcohol consumption during pregnancy in Europe are largely lacking. This explorative study aimed to estimate and compare BMI growth curves of children from seven European countries and to investigate whether associations between early life factors and BMI growth during childhood differ between countries using recent data from a large cohort. This may help to understand the inter-country heterogeneity of BMI growth and the observed differences in later obesity prevalence.

## Materials and Methods

The IDEFICS (Identification and Prevention of Dietary- and Lifestyle-Induced Health Effects in Children and Infants) cohort is a multi-centre population-based study aiming to investigate and prevent the causes of diet- and lifestyle-related diseases in children aged 2.0 to 9.9 years. The baseline survey (T0) was conducted from September 2007 to May 2008 in eight European countries (Belgium, Cyprus, Estonia, Germany, Hungary, Italy, Sweden, Spain); more than 31,500 children were invited and 16,228 of them participated and fulfilled the inclusion criteria of the IDEFICS study. Children were approached via schools and kindergartens to facilitate equal enrolment of all social groups. The survey included interviews with parents concerning lifestyle habits and dietary intakes as well as anthropometric measurements and examinations of the children. All measurements were performed according to standardised procedures in all eight countries. Details on the design and objectives of the study can be obtained from Ahrens et al. [30,31]. A follow-up survey (T1) was conducted in 2009/2010 applying the same standardised assessments where 13,596 children aged 4.0–11.9 years were enrolled; 11,041 of them participated already at T0.

### Ethics statement

We certify that all applicable institutional and governmental regulations concerning the ethical use of human volunteers were followed during this research, and that the IDEFICS project passed the Ethics Review process of the Sixth Framework Programme (FP6) of the European Commission. Ethical approval was obtained from the relevant local or national ethics committees by each of the eight study centers, namely from the Ethics Committee of the University Hospital Ghent (Belgium), the National Bioethics Committee of Cyprus (Cyprus), the Tallinn Medical Research Ethics Committee of the National Institutes for Health Development (Estonia), the Ethics Committee of the University Bremen (Germany), the Scientific and Research Ethics Committee of the Medical Research Council Budapest (Hungary), the Ethics Committee of the Health Office Avellino (Italy), the Ethics Committee for Clinical Research of Aragon (Spain), and the Regional Ethical Review Board of Gothenburg (Sweden). All parents or legal guardians of the participating children gave written informed consent to data collection, examinations, collection of samples, subsequent analysis and storage of personal data and collected samples. Additionally, each child gave oral consent after being orally informed about the modules by a study nurse immediately before every examination using a simplified text. This procedure was chosen due to the young age of the children. The oral consenting process was not further documented, but it was subject to central and local training and quality control procedures of the study. Study participants and their parents/legal guardians could consent to single components of the study while abstaining from others. All procedures were approved by the above-mentioned Ethics Committees.

### Anthropometric measurements

Height [cm] of the children was measured to the nearest 0.1 cm with a calibrated stadiometer (Seca 225 stadiometer, Birmingham, UK), body weight [kg] was measured in fasting state in light underwear on a calibrated scale accurate to 0.1 kg (Tanita BC 420 SMA, Tanita Europe GmbH, Sindelfingen, Germany). BMI was calculated as weight [kg] divided by height [m] squared. The BMI values at last follow-up (current BMI) were also converted to age- and sex-specific z-scores according to the extended IOTF criteria [32]. Birth records and records of routine child visits or registry data (Sweden) including additional height/weight measurements throughout childhood were collected in seven out of the eight IDEFICS countries (Belgium, Cyprus, Germany, Hungary, Italy, Spain and Sweden; up to 33 measures per child) and linked to the survey data. Information was supplemented by parentally reported birth weights and lengths. These reported data were used if measurements of birth length/weight were not available from the health record data.

### Covariates

Covariates were selected based on previous literature focussing on early life factors that were suggested to affect BMI growth starting from birth. Furthermore, only covariates being almost time-invariant were considered. For instance, physical activity was not considered as it is strongly time-varying and such information was not available for the health record data. Unless otherwise stated, covariate information was obtained from parentally reported questionnaires completed at the T0 and T1 survey. Questions on pregnancy-related variables were posed to biologic mothers only:

#### Mother’s height in cm, weight in kg and BMI in kg/m^2^

Information on height and weight assessed at either T0 (N = 998) or T1 (N = 6,746) was used. Based on the reported heights and weights, maternal BMI values were calculated dividing weight by height squared.

#### Educational level of parents

ISCED (International Standard Classification of Education, 1997) level of both parents was derived based on country-specific questionnaire information on highest educational attainment of the parents. ISCED levels were classified in three categories (ISCED level 0,1,2: low; ISCED level 3,4: medium; ISCED level 5,6: high).

#### Gestational age of new-born at delivery

A binary indicator was constructed for children delivered at term vs. children born pre-term (≤ 37^th^ gestational week), information was either obtained from birth records (N = 1,313), or, if not available, from parental questionnaire data (N = 6,331).

#### Gestational weight gain (kg)

Information was obtained from parental questionnaires.

#### Mother’s age at delivery (years)

Information was obtained from maternity cards (N = 212), or, if not available, from parental questionnaires (N = 7,432).

#### Frequency of smoking and alcohol consumption of mother during pregnancy

For alcohol consumption, answer categories ranged from ‘Never‘, ‘Once a month or less often’, ‘Several occasions a month’ to ‘Several occasions a week’. To enhance stability of model estimates, only a binary indicator for alcohol consumption during pregnancy (‘Never/once a month or less’ vs. at least ‘Several occasions a month’) was included in the later models. Analogously, the original smoking categories were ‘Never’, ‘Rarely, at max once a month’, ‘Several occasions a week’, ‘Daily’ but were dichotomised into ‘Never/rarely’ vs. ‘At least several occasions a week’).

#### Breast feeding duration

Starting and ending months of exclusive breast feeding and breast feeding combinations were used to derive the total breast feeding duration which was classified in ‘Not breast fed’, ‘1-<4 months’ or ‘≥4 months’ to differentiate between parents (not) complying with common breast feeding recommendations.

Previous research has shown good validity and repeatability of maternally reported birth characteristics and pregnancy-related events [33,34], also for the early life factors assessed in IDEFICS [35].

### Analysis dataset

**Fig 1:**
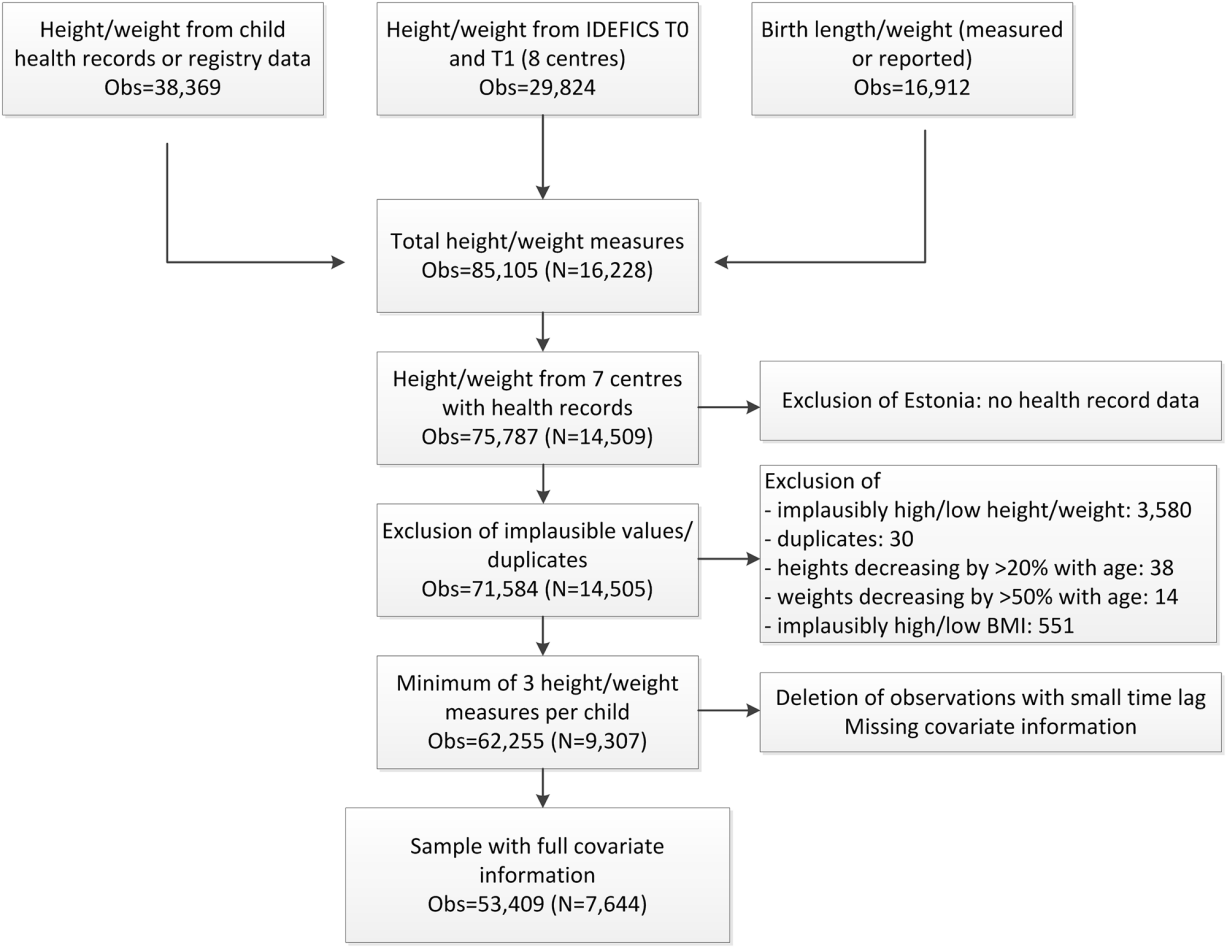
Data flow chart (Obs: number of repeated height/weight measurements; N: number of study subjects).

The flow chart (Fig 1) shows the numbers of height and weight measurements available from the different sources and summarises the exclusion process leading to the final analysis dataset.

The present analysis included only data from seven out of the eight IDEFICS countries because Estonia did not collect records of routine child visits and hence provided no information on early BMI growth. In total, 75,787 measurements corresponding to 14,509 children from the remaining seven countries were available. Numbers of measurements per child as well as time points of measurements differed between children. Implausible height/weight measurements above or below the age- and sex-specific mean +/- 4 standard deviations (SD) were excluded (N = 3,580) as well as 30 duplicate observations. Heights decreasing by more than 20% (N = 38) or weights decreasing by more than 50% (N = 14) over time were excluded. Afterwards, BMI values +8 SD above or -4SD below the mean were set to missing to exclude implausible height/weight combinations (N = 551). The final dataset included only children with a minimum of 3 repeated measurements on height and weight to achieve sufficient model stability. To account for collinearity of measurements taken closely in time, a minimum time lag of 1 month (for measurements taken below 6 months of age), 2 months (measurements between 6 months to 1.5 years) or 3 months (measurements > 1.5 years), respectively, was set. Measurements taken closer in time were randomly deleted (N = 6,794). Exclusion of children with missing covariate information led to a final analysis sample of 7,644 children with a total of 53,409 growth measurements. The average number of repeated measurements per child was 7 (median: 6, interquartile range: 3–9). In S1 File the numbers of children with 3, 4, 5, etc. available measurements (see Table A in S1 File) as well as the numbers of measurements and mean sample BMI values by age group and country are displayed (Table B in S1 File).

Percentages of overweight/obese children (23.2% vs. 20.4%), boys (53.2% vs. 51.0%) and parents being in the highest ISCED category (40.2% vs. 37.3%) were slightly higher in the present sample compared to the original IDEFICS sample.

## Statistical Analysis

BMI growth trajectories were modelled using fractional polynomial multi-level models with two levels (measurement occasion within individual) allowing individuals to have different intercepts and age-effects, i.e. their own growth trajectory. These models can easily handle unbalanced data with different numbers of repeated measures per child, as well as assessments at different time points. Moreover, such models allow for change in scale and variance of the growth measures over time [36].

All fractional polynomials with up to three powers of age out of the following powers (-2, -1, -0.5, log, square root, 1,2,3) were estimated to identify the best-fitting models for BMI growth. As age must be strictly positive when using fractional polynomials [37], a constant of 0.001 was added to age at birth. All models were estimated stratified by country. Based on the Bayesian Information Criterion (BIC) the models with the best fit for the majority of countries (and at least among the top three for the other countries) were selected to estimate children’s BMI growth. This procedure enabled comparability of subsequent country-specific model estimates. The best-fitting fractional polynomial was that with the following powers of age:

$$BMI_{i,j}=\left(\beta_{0}+u_{i,0}+\;\varepsilon_{i,j}\right)+\left(\beta_{1}+u_{i,1}\right)age_{i,j}^{1}+\left(\beta_{2}+u_{i,2}\right)age_{i,j}^{2}+\;(\beta_{3}+\;u_{i,3})\text{log}\left(age_{i,j}\right)$$

where *β*_0_, *β*_1_, *β*_2_, *β*_3_ denote the fixed intercept and slopes,*u*_*i*,0_,*u*_*i*,1_,*u*_*i*,2_,*u*_*i*,3_ denote the random intercepts and slopes and *ε*_*i,j*_ denotes the error term with *i = 1*,*…n* and *j = 1*,*…n*_*i*_ (*n*: number of subjects; *n*_*i*_: number of measurements of subject *i*).

In a first step, growth models for BMI were estimated stratified by country including only a basic adjustment (sex, gestational age at birth (pre-term vs. at term) and interactions of these with the age terms). A binary indicator for measured vs. reported birth length/weight was added to the model to adjust for differential measurement error. A formal description of this basic growth model is given in Text A in S1 File.

In a second step, single covariates and their interactions with age were added to the basic model: binary indicator variables for smoking during pregnancy (yes vs. no) and alcohol consumption during pregnancy (yes vs. no), categorical variables for breast feeding duration (reference: ≥ 4 months) and maximum ISCED level of parents (reference: ISCED level 5,6), continuous variables for maternal age at birth (centred at 30 years), gestational weight gain (centred at 10 kg) and maternal BMI (centred at 20 kg/m^2^). All covariates and interaction terms that were associated with BMI growth at the 5% level in at least one country or main effects, if a corresponding interaction term was significant, were included in the fully-adjusted model. Main effects can be interpreted as effects of the covariates on BMI at birth (except for cases where log age interaction is involved), covariate interactions with the age terms indicate the changes in the effects during childhood.

As breast feeding cannot affect BMI at birth, no main effect was included here in the model. Continuous variables were centred to ensure meaningful interpretations of effect estimates.

99% confidence intervals (CI) were used (rather than the more usual 95%) to account at least partially for multiple testing. All analyses were performed using SAS^®^ statistical software version 9.3 (SAS Institute, Inc., Cary, NC).

## Results

### Description of the analysis group

At the last follow-up (mean age: 7.9 years; range 3.9 to 11.9), the highest prevalence of overweight and obesity was observed in Italy (28.9% overweight, 21.1% obese) followed by Cyprus and Spain (Table 1). Belgium (8.1% overweight, 1.6% obese) and Sweden (10.5% overweight, 1.8% obese) exhibited the lowest prevalence. In Sweden and Hungary more than 67% of the children were breast fed for at least four months compared with only 21.6% and 18.5% in Cyprus and Italy, respectively. The highest proportion of mothers smoking during pregnancy was found in Spain (22.3%) and Germany (19.8%), the highest proportion of mothers consuming alcohol during pregnancy was observed in Belgium (11.3%). The mean age of mothers at birth was highest in Spain (32.6 y) and Sweden (31.0 y); gestational weight gain was low in Spain (11.6 kg) compared to the mean across all countries (13.9 kg). Both mothers’ and fathers were shortest in Italy and Spain, mean maternal BMI was highest in Germany and Italy, paternal BMI was highest in Hungary and Italy.

**Table 1:** Description of the study population of children aged 7.9 years on average at last follow-up by country (numbers and percentages for categorical variables; mean values and standard deviations (SD) for continuous variables).

	Belgium (N = 948)		Cyprus (N = 695)		Germany (N = 1,495)		Hungary (N = 826)		Italy (N = 1,216)		Spain (N = 1,070)		Sweden (N = 1,394)		Total (N = 7,644)	
	N	%	N	%	N	%	N	%	N	%	N	%	N	%	N	%
Thin*	120	12.7	65	9.4	146	9.8	134	16.2	51	4.2	61	5.7	158	11.3	735	9.6
Normal weight*	736	77.6	423	60.9	1083	72.4	535	64.8	557	45.8	741	69.3	1064	76.3	5,139	67.2
Overweight*	77	8.1	137	19.7	205	13.7	109	13.2	351	28.9	217	20.3	147	10.5	1,243	16.3
Obese*	15	1.6	70	10.1	61	4.1	48	5.8	257	21.1	51	4.8	25	1.8	527	6.9
Boys	521	55.0	386	55.5	777	52	440	53.3	659	54.2	582	54.4	704	50.5	4,069	53.2
Girls	427	45.0	309	44.5	718	48	386	46.7	557	45.8	488	45.6	690	49.5	3,575	46.8
Delivery at term	891	94.0	667	96.0	1379	92.2	796	96.4	1159	95.3	1002	93.6	1327	95.2	7,221	94.5
Pre-term delivery	57	6.0	28	4.0	116	7.8	30	3.6	57	4.7	68	6.4	67	4.8	423	5.5
Not breastfed	419	44.2	291	41.9	489	32.7	69	8.4	226	18.6	230	21.5	117	8.4	1,841	24.1
1-<4 month breast feeding	273	28.8	254	36.5	423	28.3	199	24.1	765	62.9	244	22.8	339	24.3	2,497	32.7
> = 4 month breast feeding	256	27.0	150	21.6	583	39.0	558	67.6	225	18.5	596	55.7	938	67.3	3,306	43.2
Alcohol No	841	88.7	685	98.6	1488	99.5	820	99.3	1171	96.3	1023	95.6	1388	99.6	7,416	97.0
Yes	107	11.3	10	1.4	7	0.5	6	0.7	45	3.7	47	4.4	6	0.4	228	3.0
Smoking No	865	91.2	669	96.3	1199	80.2	790	95.6	1127	92.7	831	77.7	1331	95.5	6,812	89.1
Yes	83	8.8	26	3.7	296	19.8	36	4.4	89	7.3	239	22.3	63	4.5	832	10.9
ISCED level 0,1,2	22	2.3	20	2.9	274	18.3	11	1.3	233	19.2	73	6.8	11	0.8	644	8.4
ISCED level 3,4	465	49.1	278	40.0	893	59.7	408	49.4	762	62.7	417	39.0	395	28.3	3,618	47.3
ISCED level 5,6	461	48.6	397	57.1	328	21.9	407	49.3	221	18.2	580	54.2	988	70.9	3,382	44.2
	**Mean**	**SD**	**Mean**	**SD**	**Mean**	**SD**	**Mean**	**SD**	**Mean**	**SD**	**Mean**	**SD**	**Mean**	**SD**	**Mean**	**SD**
Child’s age at follow-up	7.7	1.6	8.3	1.4	7.6	2.0	8.4	1.8	8.0	1.9	7.7	1.8	7.7	2.0	7.9	1.0
Mother's age at birth [y]	29.2	4.2	28.3	5.1	29.2	5.4	28.0	4.2	30.0	4.9	32.6	3.9	31.0	4.4	29.9	4.8
Gestational weight gain [kg]	13.8	5.1	14.4	5.2	15.0	6.0	15.0	5.7	13.0	5.0	11.6	4.4	14.9	5.5	13.9	5.5
Mother's height [cm]	167	6.0	164	6.1	167	6.6	166	6.1	163	5.7	163	6	167	6	166	6.3
Mother's weight [kg]	65.4	11.0	61.6	11	70.0	15	66.0	14	66.0	12	61.9	10	66.7	12	65.8	13
Mother's BMI	23.6	3.9	22.9	4.0	25.0	4.8	24.0	4.6	25.0	4.5	23.2	3.6	23.8	4.1	24.0	4.3

*BMI categories according to extended IOTF criteria [32] classified at last follow-up measurement

### BMI trajectories

**Fig 2:**
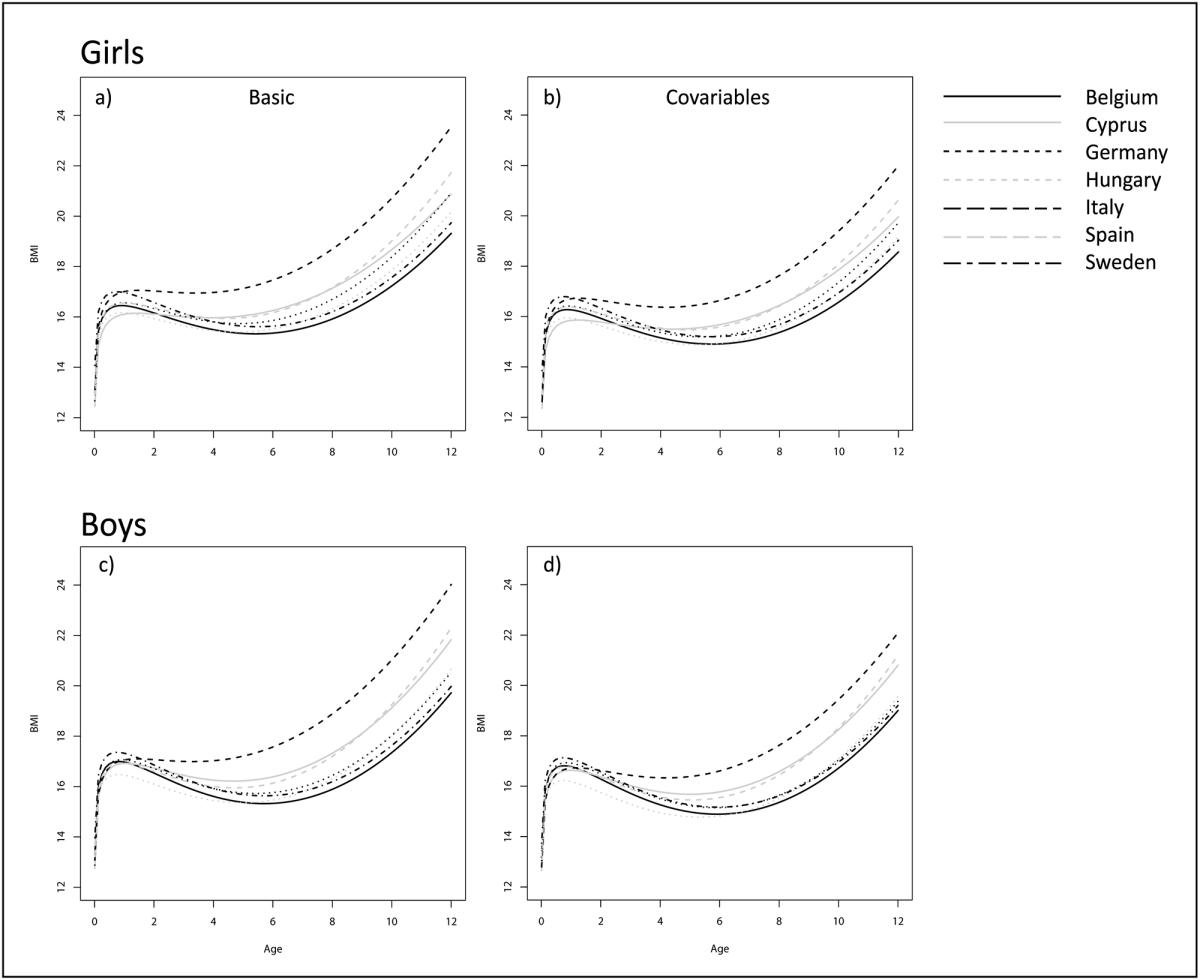
BMI growth trajectories estimated based on fractional polynomial models by country and sex comparing the basic (a,c) and covariate-adjusted model (b,d) in the respective subgroup; all continuous covariates were set to 0 (i.e. to the centred values) and categorical variables were set to the reference category.

Fig 2 shows the BMI development from birth to <12 years of age for the seven countries comparing the estimates of the basic and covariate-adjusted model by sex. Corresponding estimated marginal mean BMIs and 99% confidence intervals for one-year age groups are displayed in Table 2 for the basic and covariate-adjusted models. Because estimates obtained based on fractional polynomial models are known to be less precise at the tails [36], estimated values for the ages 0 and 12 years are not shown in Table 2 to avoid misleading conclusions.

**Table 2:** Estimated marginal mean values for BMI at ages 1 to 11 years and 99% confidence intervals for the basic and covariate-adjusted model stratified by sex. Covariates are held constant (centred values for continuous variables, reference category for categorical variables).

		Belgium			Cyprus			Germany			Hungary			Italy			Spain			Sweden		
**Basic BMI model**	**Age in years**	**Mean**	**LCI**	**UCI**	**Mean**	**LCI**	**UCI**	**Mean**	**LCI**	**UCI**	**Mean**	**LCI**	**UCI**	**Mean**	**LCI**	**UCI**	**Mean**	**LCI**	**UCI**	**Mean**	**LCI**	**UCI**
**Boys**	1	17.0	16.8	17.1	16.9	16.6	17.2	17.1	17.0	17.2	16.5	16.2	16.7	17.1	16.8	17.3	16.9	16.8	17.1	17.3	17.2	17.4
	2	16.5	16.4	16.7	16.7	16.5	17.0	16.7	16.6	16.8	16.1	15.9	16.3	17.1	16.9	17.3	16.6	16.5	16.7	16.9	16.8	16.9
	3	16.0	15.9	16.1	16.4	16.2	16.7	16.3	16.2	16.4	15.7	15.6	15.9	17.0	16.8	17.2	16.2	16.1	16.4	16.3	16.2	16.4
	4	15.6	15.5	15.7	16.2	16.0	16.5	15.9	15.8	16.0	15.4	15.3	15.6	17.0	16.8	17.2	16.0	15.9	16.1	15.9	15.8	16.0
	5	15.4	15.3	15.5	16.2	16.0	16.4	15.7	15.6	15.8	15.4	15.2	15.5	17.2	17.0	17.4	16.0	15.8	16.1	15.7	15.6	15.8
	6	15.3	15.2	15.5	16.4	16.1	16.6	15.7	15.6	15.9	15.5	15.3	15.6	17.5	17.3	17.8	16.2	16.0	16.3	15.6	15.5	15.8
	7	15.5	15.4	15.7	16.7	16.5	17.0	16.0	15.8	16.1	15.8	15.6	16.0	18.1	17.9	18.3	16.6	16.4	16.7	15.8	15.7	15.9
	8	15.9	15.7	16.1	17.3	17.0	17.6	16.4	16.2	16.6	16.3	16.1	16.5	18.8	18.6	19.1	17.2	17.0	17.4	16.2	16.1	16.4
	9	16.5	16.3	16.7	18.1	17.7	18.4	17.1	16.8	17.3	17.0	16.8	17.3	19.8	19.5	20.1	18.1	17.9	18.4	16.8	16.6	17.0
	10	17.4	17.1	17.6	19.1	18.7	19.5	18.0	17.7	18.2	18.0	17.7	18.3	20.9	20.6	21.3	19.3	19.0	19.6	17.7	17.4	17.9
	11	18.4	18.1	18.8	20.3	19.8	20.8	19.1	18.8	19.4	19.2	18.8	19.6	22.3	21.9	22.8	20.7	20.3	21.1	18.7	18.4	19.0
**Girls**	1	16.4	16.3	16.6	16.1	15.7	16.5	16.6	16.5	16.7	16.2	16.0	16.4	17.0	16.8	17.2	16.5	16.4	16.7	17.0	16.9	17.1
	2	16.2	16.0	16.3	16.1	15.8	16.4	16.3	16.2	16.4	15.9	15.8	16.1	17.0	16.8	17.2	16.4	16.2	16.5	16.6	16.5	16.7
	3	15.8	15.7	15.9	16.0	15.7	16.3	16.0	15.9	16.1	15.6	15.5	15.8	17.0	16.8	17.1	16.1	16.0	16.2	16.1	16.0	16.2
	4	15.5	15.4	15.6	16.0	15.7	16.2	15.8	15.7	15.9	15.5	15.3	15.6	17.0	16.8	17.2	16.0	15.8	16.1	15.8	15.7	15.9
	5	15.3	15.2	15.5	16.0	15.8	16.3	15.7	15.6	15.9	15.4	15.2	15.6	17.1	16.9	17.3	16.0	15.8	16.1	15.6	15.5	15.7
	6	15.4	15.2	15.5	16.3	16.0	16.5	15.9	15.7	16.0	15.5	15.3	15.7	17.4	17.2	17.7	16.2	16.0	16.3	15.6	15.5	15.8
	7	15.6	15.4	15.7	16.6	16.3	16.9	16.2	16.0	16.3	15.8	15.6	16.0	18.0	17.7	18.2	16.6	16.4	16.8	15.8	15.7	16.0
	8	15.9	15.7	16.1	17.1	16.8	17.5	16.7	16.5	16.9	16.3	16.1	16.5	18.7	18.4	18.9	17.2	17.0	17.4	16.2	16.1	16.4
	9	16.5	16.3	16.7	17.8	17.4	18.2	17.4	17.2	17.6	17.0	16.7	17.3	19.6	19.2	19.9	18.0	17.7	18.3	16.8	16.6	17.0
	10	17.2	16.9	17.5	18.7	18.2	19.1	18.4	18.1	18.6	17.9	17.5	18.2	20.7	20.3	21.0	19.0	18.7	19.3	17.6	17.4	17.8
	11	18.2	17.8	18.6	19.7	19.1	20.3	19.5	19.2	19.9	18.9	18.5	19.3	22.0	21.5	22.4	20.3	19.8	20.7	18.6	18.3	18.9
**Covariate adjusted BMI model**	**Age in years**	**Mean**	**LCI**	**UCI**	**Mean**	**LCI**	**UCI**	**Mean**	**LCI**	**UCI**	**Mean**	**LCI**	**UCI**	**Mean**	**LCI**	**UCI**	**Mean**	**LCI**	**UCI**	**Mean**	**LCI**	**UCI**
**Boys**	1	16.8	16.6	16.9	16.7	16.3	17.0	16.9	16.8	17.1	16.2	16.0	16.4	16.7	16.5	17.0	16.2	16.0	16.4	17.1	17.0	17.2
	2	16.3	16.1	16.4	16.4	16.1	16.7	16.5	16.3	16.6	15.8	15.5	16.0	16.7	16.4	16.9	15.8	15.5	16.0	16.6	16.5	16.7
	3	15.7	15.6	15.9	16.0	15.8	16.3	15.9	15.8	16.1	15.3	15.1	15.5	16.5	16.2	16.8	15.3	15.1	15.5	16.0	15.9	16.1
	4	15.3	15.1	15.4	15.8	15.5	16.1	15.5	15.3	15.7	15.0	14.8	15.2	16.4	16.1	16.7	15.0	14.8	15.2	15.6	15.4	15.7
	5	15.0	14.8	15.1	15.7	15.4	16.0	15.2	15.0	15.4	14.8	14.6	15.0	16.5	16.2	16.9	14.8	14.6	15.0	15.3	15.1	15.4
	6	14.9	14.7	15.1	15.8	15.5	16.1	15.1	14.9	15.4	14.8	14.6	15.1	16.8	16.4	17.2	14.8	14.6	15.1	15.2	15.0	15.3
	7	15.0	14.8	15.2	16.1	15.8	16.5	15.3	15.0	15.5	15.1	14.8	15.3	17.2	16.8	17.6	15.1	14.8	15.3	15.3	15.1	15.5
	8	15.3	15.1	15.6	16.6	16.2	17.0	15.6	15.3	15.9	15.5	15.2	15.8	17.8	17.3	18.3	15.5	15.2	15.8	15.7	15.5	15.8
	9	15.9	15.6	16.2	17.4	16.9	17.8	16.2	15.9	16.5	16.2	15.8	16.5	18.7	18.1	19.2	16.2	15.8	16.5	16.2	16.0	16.4
	10	16.7	16.3	17.0	18.3	17.8	18.8	17.0	16.6	17.4	17.1	16.7	17.5	19.7	19.1	20.4	17.1	16.7	17.5	17.0	16.7	17.3
	11	17.7	17.3	18.1	19.5	18.9	20.1	18.0	17.6	18.5	18.2	17.7	18.7	21.0	20.2	21.7	18.2	17.7	18.7	18.0	17.7	18.3
**Girls**	1	16.3	16.1	16.4	15.9	15.5	16.3	16.4	16.3	16.6	15.9	15.7	16.2	16.7	16.4	17.0	15.9	15.7	16.2	16.8	16.6	16.9
	2	15.9	15.8	16.1	15.8	15.4	16.1	16.1	15.9	16.2	15.6	15.4	15.9	16.6	16.4	16.9	15.6	15.4	15.9	16.3	16.2	16.5
	3	15.5	15.3	15.7	15.6	15.3	15.9	15.7	15.5	15.9	15.3	15.1	15.5	16.5	16.2	16.7	15.3	15.1	15.5	15.9	15.7	16.0
	4	15.2	15.0	15.3	15.5	15.2	15.8	15.4	15.2	15.6	15.0	14.8	15.2	16.4	16.1	16.7	15.0	14.8	15.2	15.5	15.4	15.6
	5	15.0	14.8	15.1	15.6	15.2	15.9	15.2	15.0	15.4	14.9	14.7	15.1	16.4	16.1	16.8	14.9	14.7	15.1	15.3	15.1	15.4
	6	14.9	14.7	15.1	15.7	15.4	16.1	15.2	15.0	15.5	14.9	14.7	15.2	16.6	16.2	17.0	14.9	14.7	15.2	15.2	15.1	15.4
	7	15.1	14.8	15.3	16.0	15.7	16.4	15.5	15.2	15.7	15.2	14.9	15.4	17.0	16.6	17.5	15.2	14.9	15.4	15.4	15.2	15.5
	8	15.4	15.1	15.6	16.5	16.1	16.9	15.9	15.6	16.2	15.6	15.3	15.9	17.6	17.1	18.1	15.6	15.3	15.9	15.7	15.5	15.9
	9	15.9	15.6	16.2	17.1	16.7	17.6	16.5	16.2	16.9	16.2	15.8	16.5	18.4	17.9	19.0	16.2	15.8	16.5	16.2	16.0	16.5
	10	16.6	16.2	17.0	17.9	17.4	18.5	17.4	17.0	17.8	16.9	16.5	17.4	19.4	18.8	20.1	16.9	16.5	17.4	17.0	16.7	17.2
	11	17.5	17.1	17.9	18.9	18.2	19.6	18.4	18.0	18.9	17.9	17.4	18.4	20.6	19.9	21.3	17.9	17.4	18.4	17.9	17.6	18.2

LCI: lower confidence interval; UCI: upper confidence interval

Starting from infancy, the basic model revealed large between-country differences (Fig 2a and 2d) with Italy showing the steepest BMI growth curve followed by Cyprus and Spain. Conversely, Belgium and Hungary showed the flattest BMI growth curves and lowest BMI in mid childhood. Mean BMI values at 3 years of age and all subsequent ages were significantly higher in Italy compared to the other countries in the basic model (Table 2; top). For instance, in Italy the estimated mean BMI at age 11 years is 22.3 in boys and 22.0 in girls, respectively, compared to range of 18.4 (Belgium) to 20.3 (Spain) in boys and 18.2 (Belgium) to 19.8 (Spain) in girls in the other countries. After adjustment for covariates (Fig 2b and 2d; Table 2 middle), the country-specific growth curves became more similar, but some differences persisted.

**Fig 3:**
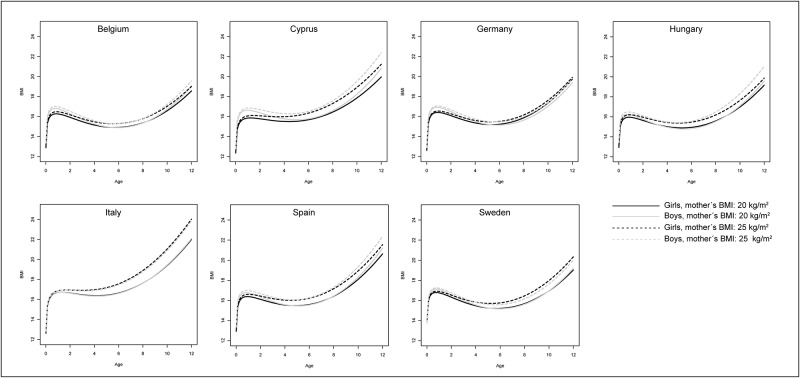
Predicted BMI growth trajectories of children having a mother with a BMI of 20 kg/m^2^ (solid) vs. with a BMI of 25 kg/m^2^ (dashed) by sex (girls: black; boys: grey) and country; all other covariates were set to a constant value (continuous covariates were set to 0 (i.e. to the value used for centring), categorical variables were set to the reference category).

**Fig 4:**
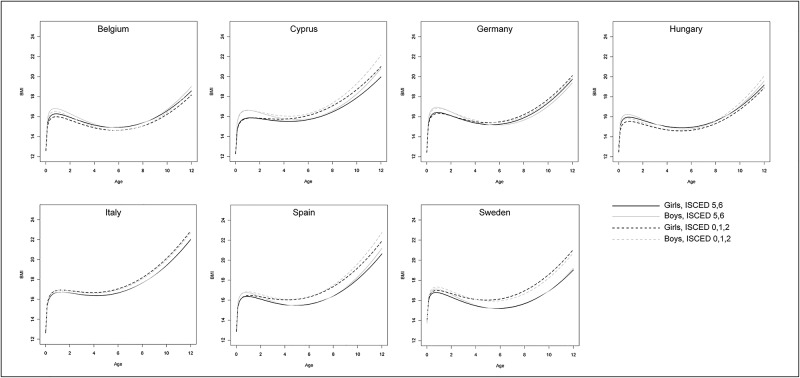
Predicted BMI growth trajectories of children having parents with high (ISCED 5,6; solid) vs. low educational level (ISCED 0,1,2; dashed) by sex (girls: black; boys: grey) and country; all other covariates were set to a constant value (continuous covariates were set to 0 (i.e. to the value used for centring), categorical variables were set to the reference category).

The parameter estimates with 99% CI of the final covariate-adjusted BMI growth model are displayed in Table 3; results of the basic model are presented in Table C in S1 File. The effect estimates are difficult to interpret as main effects need to be interpreted together with their age-interactions [36], and are best visualized in a figure. For this reason, BMI trajectories were plotted according to single covariates keeping all other covariates constant (see Figs 3 and 4 and Figs A to D in S1 File). In the following we further describe how to interpret the effect estimates using the association between maternal BMI (centred at 20 kg/m^2^) and children’s’ BMI growth in Italy as an example: The estimated main effect is 0.00 kg/m^2^ and the age*maternal BMI interaction is 0.03 kg/m^2^/yr, i.e. for 1 unit increase in age and 1 unit increase in maternal BMI, the BMI of the child increases by 0.03 units (0.00 + 0.03). For instance, if the value of maternal BMI was 25 (i.e. the centred value is 25–20 = 5), a child’s BMI will be higher by (25–20)*0.00 + 4*(25–20)*0.03 = 0.6 units at age 4 and higher by (25–20)*0.00 + 10*(25–20)*0.03 = 1.5 units at age 10 compared to a child with a mother having a BMI of 20 kg/m^2^.

**Table 3:** Effect estimates and 99% confidence intervals of the covariate-adjusted growth models for BMI for the seven countries. Continuous variables were centred to receive meaningful effect estimates (mother’s BMI centred at 20 kg/m^2^, mother’s age at birth centred at 30 years, gestational weight gain centred at 10 kg).

BMI model		Belgium			Cyprus			Germany			Hungary			Italy			Spain			Sweden		
		ß	LCI	UCI	ß	LCI	UCI	ß	LCI	UCI	ß	LCI	UCI	ß	LCI	UCI	ß	LCI	UCI	ß	LCI	UCI
**Intercept**		17.51	17.27	17.74	16.76	16.18	17.34	17.73	17.54	17.92	17.07	16.72	17.42	17.77	17.38	18.15	17.58	17.35	17.81	18.02	17.88	18.17
**Age**		-1.34	-1.44	-1.24	-1.00	-1.22	-0.77	-1.43	-1.51	-1.36	-1.24	-1.38	-1.09	-1.16	-1.33	-1.00	-1.31	-1.40	-1.22	-1.38	-1.43	-1.32
**Age**^**2**^		0.10	0.09	0.11	0.09	0.07	0.10	0.11	0.11	0.12	0.10	0.09	0.11	0.11	0.10	0.12	0.11	0.11	0.12	0.11	0.10	0.11
**log(age)**		1.00	0.94	1.07	0.95	0.79	1.12	1.11	1.06	1.15	0.90	0.80	0.99	1.11	1.02	1.21	1.01	0.95	1.06	0.90	0.87	0.94
**Boys**		0.77	0.48	1.06	1.16	0.44	1.88	0.72	0.52	0.91	0.42	-0.03	0.87	0.02	-0.45	0.49	0.58	0.32	0.85	0.53	0.36	0.71
**Girls**		0.00	.	.	0.00	.	.	0.00	.	.	0.00	.	.	0.00	.	.	0.00	.	.	0.00	.	.
Age*sex	**Boys**	-0.28	-0.41	-0.15	-0.40	-0.68	-0.12	-0.22	-0.31	-0.13	-0.18	-0.38	0.01	0.01	-0.20	0.22	-0.23	-0.35	-0.11	-0.22	-0.29	-0.14
	**Girls**	0.00	.	.	0.00	.	.	0.00	.	.	0.00	.	.	0.00	.	.	0.00	.	.	0.00	.	.
**(Age**^**2**^)*sex	**Boys**	0.02	0.01	0.03	0.03	0.01	0.05	0.01	0.00	0.02	0.02	0.00	0.03	0.00	-0.01	0.02	0.02	0.01	0.03	0.01	0.01	0.02
	**Girls**	0.00	.	.	0.00	.	.	0.00	.	.	0.00	.	.	0.00	.	.	0.00	.	.	0.00	.	.
log(Age)*sex	**Boys**	0.10	0.03	0.18	0.19	0.02	0.35	0.11	0.06	0.16	0.02	-0.09	0.13	-0.03	-0.14	0.08	0.06	-0.01	0.13	0.14	0.10	0.18
	**Girls**	0.00	.	.	0.00	.	.	0.00	.	.	0.00	.	.	0.00	.	.	0.00	.	.	0.00	.	.
**Delivery status**	**Pre-term**	0.20	-0.38	0.78	-1.49	-3.46	0.49	0.10	-0.27	0.47	0.77	-0.16	1.69	-0.06	-1.09	0.97	0.76	0.26	1.26	0.17	-0.24	0.58
	**At term**	0.00	.	.	0.00	.	.	0.00	.	.	0.00	.	.	0.00	.	.	0.00	.	.	0.00	.	.
Age*delivery	**Pre-term**	-0.27	-0.54	-0.00	0.31	-0.47	1.10	-0.23	-0.41	-0.06	-0.53	-0.95	-0.11	-0.32	-0.80	0.17	-0.53	-0.77	-0.29	-0.28	-0.47	-0.10
	**At term**	0.00	.	.	0.00	.	.	0.00	.	.	0.00	.	.	0.00	.	.	0.00	.	.	0.00	.	.
**(Age**^**2**^)*delivery	**Pre-term**	0.01	-0.01	0.04	-0.01	-0.07	0.04	0.02	0.00	0.03	0.04	0.01	0.07	0.03	0.00	0.07	0.04	0.01	0.06	0.02	0.00	0.04
	**At term**	0.00	.	.	0.00	.	.	0.00	.	.	0.00	.	.	0.00	.	.	0.00	.	.	0.00	.	.
log(Age)*delivery	**Pre-term**	0.42	0.26	0.57	-0.17	-0.62	0.28	0.23	0.13	0.34	0.36	0.10	0.61	0.20	-0.04	0.45	0.60	0.45	0.74	0.48	0.36	0.61
	**At term**	0.00	.	.	0.00	.	.	0.00	.	.	0.00	.	.	0.00	.	.	0.00	.	.	0.00	.	.
**Reported measurement**	**Yes**	0.37	0.21	0.53	0.22	-0.15	0.60	-0.03	-0.25	0.19	-0.62	-0.86	-0.39	0.03	-0.14	0.21	0.09	-0.07	0.26	-0.96	-1.43	-0.50
	**No**	0.00	.	.	0.00	.	.	0.00	.	.	0.00	.	.	0.00	.	.	0.00	.	.	0.00	.	.
Log(Age)*breastfeeding	**Not breastfed**	0.02	-0.02	0.06	0.02	-0.04	0.08	0.01	-0.02	0.04	0.08	0.00	0.15	-0.02	-0.08	0.03	0.05	0.01	0.10	0.04	-0.01	0.08
	**< 4 months**	0.02	-0.03	0.06	0.01	-0.05	0.08	0.00	-0.03	0.03	0.04	-0.02	0.09	-0.03	-0.08	0.01	0.02	-0.02	0.06	0.02	-0.01	0.05
	**≥ 4 months**	0.00	.	.	0.00	.	.	0.00	.	.	0.00	.	.	0.00	.	.	0.00	.	.	0.00	.	.
**ISCED**	**Level 0,1,2**	-0.31	-0.78	0.16	-0.12	-0.76	0.52	-0.18	-0.35	-0.01	-0.45	-1.23	0.33	0.17	-0.09	0.42	-0.05	-0.34	0.23	0.12	-0.44	0.68
	**Level 3,4**	-0.05	-0.19	0.08	0.10	-0.12	0.31	-0.12	-0.25	0.01	-0.11	-0.29	0.07	0.07	-0.13	0.27	0.06	-0.08	0.21	-0.04	-0.16	0.07
	**Level 5,6**	0.00	.	.	0.00	.	.	0.00	.	.	0.00	.	.	0.00	.	.	0.00	.	.	0.00	.	.
Age*ISCED	**Level 0,1,2**	0.01	-0.10	0.12	0.09	-0.08	0.25	0.09	0.04	0.14	0.04	-0.13	0.21	0.02	-0.06	0.10	0.15	0.08	0.22	0.12	-0.01	0.24
	**Level 3,4**	0.04	0.01	0.07	-0.06	-0.12	0.00	0.04	0.00	0.08	0.01	-0.03	0.06	-0.04	-0.10	0.03	0.02	-0.02	0.06	0.02	-0.01	0.05
	**Level 5,6**	0.00	.	.	0.00	.	.	0.00	.	.	0.00	.	.	0.00	.	.	0.00	.	.	0.00	.	.
**Smoking in pregnancy: yes**	**Yes**	-0.05	-0.29	0.18	0.38	-0.24	1.00	0.01	-0.14	0.16	0.22	-0.19	0.63	-0.09	-0.46	0.28	-0.07	-0.25	0.11	0.28	0.01	0.54
**Smoking in pregnancy: no**	**No**	0.00	.	.	0.00	.	.	0.00	.	.	0.00	.	.	0.00	.	.	0.00	.	.	0.00	.	.
Log(Age)*Smoking	**Yes**	0.09	0.03	0.15	0.10	-0.05	0.25	0.03	-0.01	0.07	0.11	-0.01	0.23	0.10	0.01	0.20	0.07	0.02	0.11	0.06	0.00	0.13
	**No**	0.00	.	.	0.00	.	.	0.00	.	.	0.00	.	.	0.00	.	.	0.00	.	.	0.00	.	.
**Mother's BMI**		0.03	0.02	0.05	0.03	0.00	0.05	0.02	0.01	0.03	0.04	0.02	0.06	0.00	-0.01	0.02	0.03	0.01	0.05	0.02	0.00	0.03
Age*mother's BMI		0.01	0.00	0.01	0.02	0.01	0.02	0.01	0.01	0.01	0.01	0.01	0.02	0.03	0.02	0.03	0.02	0.02	0.03	0.01	0.01	0.01
**Mother's age at birth [y]**		-0.00	-0.02	0.01	0.01	-0.01	0.03	-0.00	-0.01	0.01	0.01	-0.01	0.04	0.00	-0.01	0.02	-0.01	-0.03	0.01	0.01	-0.01	0.02
Age*mother's age at birth [y]		0.00	-0.00	0.00	-0.01	-0.01	-0.00	0.00	0.00	0.01	-0.01	-0.01	-0.00	-0.01	-0.01	-0.00	-0.00	-0.01	0.00	-0.00	-0.01	0.00
**Gestational weight gain [kg]**		0.02	0.00	0.03	0.04	0.01	0.06	0.01	0.00	0.02	0.02	0.00	0.03	0.04	0.02	0.06	0.02	0.00	0.04	0.02	0.01	0.03
Log(age)* gestational weight gain [kg]		-0.00	-0.01	-0.00	0.00	-0.00	0.01	-0.00	-0.00	0.00	-0.00	-0.01	0.00	0.00	-0.00	0.01	-0.00	-0.00	0.00	-0.00	-0.01	-0.00

LCI: lower confidence interval; UCI: upper confidence interval

The main factor being positively associated with children’s BMI growth was maternal BMI (p<0.01 in all countries). The corresponding figure indicates that BMI growth was markedly higher in children of heavier mothers (25 kg/m^2^ vs. 20 kg/m^2^), especially in Italy and Cyprus (see Fig 3). Weight gain during pregnancy was slightly positively associated with BMI at birth in all countries, i.e. children of mothers with a higher gestational weight gain (15kg vs. 10kg) have a larger BMI at birth (Table 3 and Fig A in S1 File). The association with maternal BMI seems to be stronger in later childhood, whereas the association with gestational weight gain diminishes as children get older. With regard to the other covariates, results were less consistent between countries. BMI increased faster in children having parents with lower educational level (reference: ISCED 5,6) at an age of two years and thereafter in Cyprus, Germany, Italy, Spain and Sweden (Fig 4). However, significant associations were only found in Germany and Spain. Children delivered pre-term showed a similar or slightly lower (Cyprus, Hungary, Italy) BMI until an age of eight years compared to children delivered at term, afterwards the picture is reversed in Hungary, Italy and Sweden (see Fig B in S1 File). In Hungary and Spain, not being breast fed (reference: ≥ 4 months of breast feeding) was found to be slightly positively associated with BMI growth during childhood (interaction with log(age); see also Fig C in S1 File). In some countries there was a positive interaction between smoking during pregnancy and log(age) (see also Fig D in S1 File), but effect sizes were rather small. There was no evidence for an association of alcohol consumption during pregnancy (not included in fully-adjusted model) or maternal age at birth with BMI growth in any country.

## Discussion

In a previous study based on the IDEFICS cohort, Bammann et al investigated associations between early life factors and obesity risk in later childhood where parental BMI and GWG were the main factors being associated with childhood obesity [6]. The present investigation built up on that study addressing associations between early life factors and BMI growth starting from birth and further assessed inter-country heterogeneity in BMI growth. Inter-country differences in BMI growth were observed already during infancy and increased to childhood. Although various associations between early life factors and growth were identified and mean levels and prevalence of the factors differed between countries, early life factors explained only a minor part of the differences in BMI growth curves between countries.

In univariate analysis, breast feeding of at least 4 months was associated with lower BMI growth during childhood. However, in the fully-adjusted model, associations between breast feeding duration and BMI growth were only found in Hungary and Spain where Hungary (together with Sweden) was the country with the lowest proportion of children not being breast fed. As already suggested by Bammann et al. [6] this might be partly explained by confounding e.g. through maternal BMI. The literature provides inconclusive evidence on the association between breastfeeding and weight status during childhood and later life [5,38]. These inconsistent results with view to different study populations could partly result from differences in the operationalization of breast feeding (e.g. definition of optimal duration, (non-)distinction between exclusive and non-exclusive breast feeding), differences in confounders considered [38] but also from differences in nutrient content of formula milk [39], social patterning of breast feeding and recommendations on breast feeding duration [24,25]. Despite large inter-country differences in the prevalence of alcohol consumption and smoking during pregnancy, these factors did not explain much of the heterogeneity in BMI growth between countries. Associations between alcohol consumption during pregnancy and later obesity have been rarely addressed in previous studies but as alcohol has been shown to be associated with pre-term birth [40], this may be a link relating alcohol (mediated through pre-term birth and low gestational weight) to later obesity risk. In a recent study, maternal obesity in early pregnancy was identified as a main factor leading to an unfavourable BMI growth trajectory [4]. This is consistent with the strong associations between maternal BMI and children’s BMI trajectories observed in the present study, especially in Italy, where also the mean maternal and paternal BMI was found to be highest. The association between parental BMI and children’s BMI growth may on the one hand reflect the shared environment and lifestyle (family meals, physical activity, etc.), but on the other hand also shared genetic backgrounds. Heritability estimates for BMI have been reported to lie between 0.71 to 0.86 (fraction of total phenotypic variance of a quantitative trait attributable to genes in a specified environment) [41].

The associations observed in our study largely agree with the findings of a systematic review and meta-analysis on early life risk factors for childhood overweight [5]. For gestational weight gain, the authors reported inconclusive evidence whereas we found small positive associations with BMI at birth in all seven countries that decrease when children get older. Associations between gestational weight gain and BMI at birth were largest in Cyprus and Italy, being also the countries with the steepest BMI growth in general. This is further in line with two previous IDEFICS investigations that identified gestational weight gain as an independent predictor of overweight and body fat distribution in pre-school and school-aged children [6,42].

Proportions of parents being in the highest ISCED category markedly differed between countries. Noteworthy, the proportions of parents in the highest ISCED categories were lowest in Italy (18.2%) and highest in Sweden (70.9%) being the countries with the highest and second-lowest overweight/obesity prevalence, respectively. These differences in proportions of educational levels might also contribute to differences in other lifestyle factors (e.g. diet, physical activity) or health-related behaviours. For instance, children with higher-educated mothers and fathers were shown to be more likely to have a healthy dietary pattern compared to children with lower-educated parents [43]. In the present analysis, associations between parental ISCED level and BMI growth in later childhood were found only in some countries. A previous analysis based on the IDEFICS data already showed that the strength of the association between socio-economic indicators and overweight/obesity varies across European regions being weaker in countries with a lesser degree of human development [44].

BMI growth during childhood as well as the later prevalence of overweight/obesity was largest in the Southern European countries Italy, Cyprus and Spain. Consistently, in a recent review comparing the prevalence of adult overweight between European countries, the largest prevalence was reported for Southern European countries, especially Cyprus and Spain [29]. The authors suggested that this could result from the globalisation of certain lifestyle factors which might have a negative effect on the traditional Mediterranean diet [29,45]. Also IDEFICS data showed that adherence to a Mediterranean-like diet in children is not necessarily a pattern of the Mediterranean region [46], [47]. In addition, climate and further environmental factors differing by geographic region might be important unmeasured factors that could affect BMI growth. For instance, one could assume that a very hot or cold climate might result in reduced physical activity [48,49]. Moreover, there seems to be a north-south gradient in Europe when comparing mean adult height across populations [50] which may also to some degree add (mathematically due to the inverse association) to the higher BMI growth in these countries. However, it is interesting to note that whilst we found only minor differences in height trajectories across all the European countries (Figure not shown), BMI trajectories started to diverge from the age of 3 years onwards. This may reflect the main effects of an obesogenic environment or some interaction between genetic, early life and later life environmental factors.

### Strengths and limitations

The various early life factors, the multi-centre design as well as large number of repeated measurements of height and weight during infancy and childhood are clear strengths of this study. The IDEFICS survey was setting-based and not intended to provide nationally representative samples. Although this approach enabled equal enrolment of all social groups, non-response bias resulting from over-representation of certain subgroups cannot be precluded where in particular socio-economic status is a key factor associated with participation as well as with health outcomes [51,52]. In the present study, attrition effects should play a minor role as participation in T0 and T1 was not a requirement for inclusion. The application of fractional polynomials based on mixed effects models allowed optimal use of the available data considering children with varying numbers of and time spans between repeated growth measurements under a missing at random assumption and allowed modelling of smooth functions of individual growth. One limitation of these growth models includes the imprecision of the estimates at the tails [36] such that these should be interpreted with caution. To account at least partially for multiple testing, only effect estimates with p<0.01 were considered as statistically significant in the final models. However, the explorative nature of this study should be kept in mind. Genetic and environmental factors as well as their interactions affect obesity susceptibility and potentially growth but have not been considered in this study. Also dietary intake (except breast feeding), physical activity, television watching, sleep duration, etc. may largely differ between countries and hence contribute to the observed inter-country differences. Due to the use of routine data from health records such time-varying covariates were not available throughout childhood and could hence not be accounted for. Birth weight has been shown to be an independent risk factor of childhood obesity [53] but was not considered as such in the current study as it was part of the outcome vector. As with all studies, measurement errors cannot be precluded and especially variables like smoking/alcohol during pregnancy may be prone to misreporting resulting from social desirable answer behaviour. Furthermore, standardized survey measurements of height/length and weight were supplemented by data from child health records. Previous research showed good accuracy of child health record data supporting their use in research [54]. Ethnicity or migrant status was not directly accounted for in the models, i.e. the country classification was made based on the country in which the child lived and participated in routine health checks, but not based on ethnicity of the parents. This decision was corroborated by the idea of a shared environment including shared health policies, access to food at schools, built environment and climate which all compromise unobserved factors that may influence growth.

## Conclusions

Early life factors seem to explain only a small part of the observed differences in childhood BMI trajectories between countries. Maternal BMI was the factor showing the strongest association with children’s BMI growth in the present study. Apart from genetics, changes in physical activity and nutrition behaviours caused by the obesogenic environment during the last decades are likely to play a major role in the development of overweight/obesity in children and should be investigated in future studies to better understand the observed heterogeneity in childhood growth between European countries.

## Supporting Information

S1 File The supplementary material file contains two further tables describing the study sample and measurements (Tables A, B), a formal description of the BMI growth model (Text A), the results of the basic BMI growth model (Table C) as well as four additional figures displaying associations between early life factors and BMI growth (Figs A to D). (DOCX)
