# Supplementary material for: Early Life Factors and Inter-Country Heterogeneity in BMI Growth Trajectories of European Children: The IDEFICS Study
Source: PLoS One. 2016 Feb 22;11(2):e0149268. doi: 10.1371/journal.pone.0149268 (PMC4762899; doi:10.1371/journal.pone.0149268)
Supplement: S1 File — (DOCX) [file pone.0149268.s001.docx]

**Supplementary material S1**

|  | **Children with N repeated measurements** | | | | | | | | **Total** | **Total** |
| --- | --- | --- | --- | --- | --- | --- | --- | --- | --- | --- |
| **Country / N** | **3** | **4** | **5** | **6** | **7** | **8** | **9** | **≥10** | **Children** | **Measurements** |
| **Belgium** | 546 | . | . | 6 | 6 | 7 | 25 | 358 | 948 | 5,840 |
| **Cyprus** | 624 | 1 | . | 2 | 1 | 1 | 2 | 64 | 695 | 2,732 |
| **Germany** | 160 | 22 | 53 | 100 | 178 | 336 | 386 | 260 | 1,495 | 11,646 |
| **Hungary** | 591 | 9 | 7 | 8 | 6 | 8 | 14 | 183 | 826 | 4,924 |
| **Italy** | 897 | 96 | 120 | 103 | . | . | . | . | 1,216 | 4,311 |
| **Spain** | 416 | 7 | 5 | 9 | 10 | 19 | 19 | 585 | 1,070 | 11,294 |
| **Sweden** | 30 | 8 | 8 | 34 | 108 | 294 | 476 | 436 | 1,394 | 12,662 |
| **Total** | 3,264 | 143 | 193 | 262 | 309 | 665 | 922 | 1,886 | 7,644 | 53,409 |

**Table A:** Number of children with N repeated height/weight measurements, total numbers of children and measurements by country; sample with full covariate information

|  | **Belgium (N=948; Obs=5,840)** | | | **Cyprus (N=695; Obs=2,732)** | | | **Germany (N=1,495; Obs=11,646)** | | | **Hungary (N=826; Obs=4,924)** | | |
| --- | --- | --- | --- | --- | --- | --- | --- | --- | --- | --- | --- | --- |
|  | **Obs** | **Mean** | **SD** | **Obs** | **Mean** | **SD** | **Obs** | **Mean** | **SD** | **Obs** | **Mean** | **SD** |
| **<0.1 y** | 983 | 13.5 | 1.3 | 687 | 12.8 | 1.5 | 1,616 | 13.1 | 1.4 | 839 | 12.8 | 1.5 |
| **0.1 to < 2 y** | 1,613 | 16.4 | 1.5 | 219 | 16.3 | 1.5 | 4,943 | 16.4 | 1.7 | 778 | 16.2 | 1.8 |
| **2 to <4 y** | 405 | 15.8 | 1.2 | 161 | 16.1 | 1.7 | 1,227 | 16.0 | 1.5 | 481 | 15.5 | 1.8 |
| **4 to <6 y** | 540 | 15.5 | 1.4 | 336 | 16.0. | 1.9 | 1,725 | 15.8 | 1.6 | 581 | 15.2 | 1.7 |
| **6 to <8 y** | 738 | 15.6 | 1.7 | 678 | 16.7 | 2.6 | 936 | 16.1 | 2.0 | 887 | 15.9 | 2.4 |
| **8 to <10 y** | 474 | 16.2 | 2.1 | 502 | 17.9 | 3.2 | 638 | 17.1 | 2.7 | 746 | 17.1 | 2.9 |
| **10 to <12 y** | 60 | 16.6 | 2.0 | 70 | 19.1 | 3.7 | 185 | 18.5 | 3.5 | 270 | 17.8 | 3.2 |
|  | **Italy (N=1,216; Obs=4,311)** | | | **Spain (N=1,070; Obs=11,294)** | | | **Sweden (N=1,394; Obs=12,662)** | | | **All (N=7,644; Obs=53,409)** | | |
|  | **Obs** | **Mean** | **SD** | **Obs** | **Mean** | **SD** | **Obs** | **Mean** | **SD** | **Obs** | **Mean** | **SD** |
| **<0.1 y** | 1,249 | 13.1 | 1.5 | 1,128 | 13.2 | 1.4 | 1,247 | 13.9 | 1.3 | 7,749 | 13.2 | 1.5 |
| **0.1 to < 2 y** | 596 | 16.9 | 2.1 | 4,085 | 16.5 | 1.6 | 4,096 | 17.1 | 1.4 | 16,330 | 16.6 | 1.6 |
| **2 to <4 y** | 210 | 16.5 | 1.6 | 1,061 | 16.0 | 1.4 | 1,784 | 16.3 | 1.3 | 5,329 | 16.1 | 1.5 |
| **4 to <6 y** | 514 | 17.0 | 2.3 | 953 | 16.0 | 1.6 | 2,982 | 15.7 | 1.4 | 7,631 | 15.8 | 1.6 |
| **6 to <8 y** | 778 | 18.1 | 3.0 | 1,026 | 16.5 | 2.1 | 1,046 | 16.0 | 1.8 | 6,089 | 16.4 | 2.4 |
| **8 to <10 y** | 637 | 19.8 | 3.7 | 630 | 17.9 | 2.8 | 1,301 | 16.6 | 2.1 | 4,928 | 17.4 | 3.0 |
| **10 to <12 y** | 233 | 21.3 | 4.1 | 96 | 18.6 | 2.7 | 206 | 17.1 | 2.4 | 1,120 | 18.6 | 3.6 |

Table B: Numbers of BMI measurements and mean sample BMI values by age group and country

SD: standard deviation

**Text A: Formal description of the growth model**

The general growth model was defined as follows:

$${GROWTH}_{i,j}= {(\beta}_{0}+u_{i,0}+ \varepsilon_{i,j})+ {(\beta}_{1}+u_{i,1}){A1}_{i,j}+ {(\beta}_{2}+u_{i,2}){A2}_{i,j}+ {(\beta}_{3}+u_{i,3}){A3}_{i,j}+ \beta_{4}\left( {male}_{i} \right)+ {\beta_{5}\left( {male}_{i} \right){A1}_{i,j} + \beta_{6}\left( {male}_{i} \right){A2}_{i,j} +\beta_{7}\left( {male}_{i} \right){A3}_{i,j}+\beta_{8}\left( {preterm}_{i} \right)+ \beta_{9}\left( {preterm}_{i} \right){A1}_{i,j} + \beta_{10}\left( {preterm}_{i} \right){A2}_{i,j} +\beta_{11}\left( {preterm}_{i} \right){A3}_{i,j}+\beta_{12}\left( {source}_{i,j} \right),}$$

where ${GROWTH}_{i,j}$ denotes the *j*’s growth measure (height, weight or BMI, respectively) of child *i*, the fixed coefficient $\beta_{0}$ describes the average intercept for females delivered at term, $\beta_{1}$ is the average predicted change in the outcome measure for the first age variable (A1=*age* for the BMI, height and weight model), $\beta_{2}$ the average change for the second age variable (A2=age^2^ for the BMI and weight model, age^-1^ for the height model) and $\beta_{3}$the average change for the third age variable (A3=*log(age)* for BMI, *sqrt(age)* for weight and height) in female children delivered at term, $\beta_{4}$ the difference in average intercept between males and females, $\beta_{5}{,\beta}_{6}$ and $\beta_{7}$denote the difference in average slopes between males and females, $\beta_{8}$ the difference in average intercept between full-term and pre-term delivered children, $\beta_{9}{,\beta}_{10}$ and $\beta_{11}$ denote the differences in average linear slopes between full-term and pre-term delivered children, and $\beta_{12}$ describes the average difference in intercept between self-reported and routinely measured birth weights/heights. The random coefficients$u_{i,k}$, *k=1,2,3,* indicate the deviation of individual*i* from the average slope between knot points *k-1* and *k* and $u_{i0}$ describes the deviation of individual *i*’s intercept from the average intercept. An unstructured covariance matrix was modelled for the random effects, i.e. each variances/covariance could take the value that the data demand (no restrictions). The model further accounted for changes in variances of growth during childhood by defining heterogeneity by age group in the covariance structure of the measurement errors.

| **BMI model** |  | **Belgium** | | | **Cyprus** | | | **Germany** | | | **Hungary** | | | **Italy** | | | **Spain** | | | **Sweden** | | |
| --- | --- | --- | --- | --- | --- | --- | --- | --- | --- | --- | --- | --- | --- | --- | --- | --- | --- | --- | --- | --- | --- | --- |
|  |  | **ß** | **LCI** | **UCI** | **ß** | **LCI** | **UCI** | **ß** | **LCI** | **UCI** | **ß** | **LCI** | **UCI** | **ß** | **LCI** | **UCI** | **ß** | **LCI** | **UCI** | **ß** | **LCI** | **UCI** |
| **Intercept** |  | 17.64 | 17.42 | 17.85 | 16.97 | 16.40 | 17.54 | 17.78 | 17.64 | 17.93 | 17.23 | 16.91 | 17.56 | 17.95 | 17.61 | 18.29 | 17.68 | 17.48 | 17.88 | 18.19 | 18.06 | 18.31 |
| **Age** |  | -1.29 | -1.39 | -1.19 | -0.95 | -1.17 | -0.72 | -1.33 | -1.40 | -1.27 | -1.17 | -1.31 | -1.02 | -1.06 | -1.21 | -0.90 | -1.24 | -1.34 | -1.15 | -1.33 | -1.38 | -1.27 |
| **Age^2^** |  | 0.10 | 0.09 | 0.11 | 0.09 | 0.07 | 0.10 | 0.11 | 0.11 | 0.12 | 0.10 | 0.09 | 0.11 | 0.11 | 0.10 | 0.12 | 0.11 | 0.11 | 0.12 | 0.11 | 0.10 | 0.11 |
| **log(Age)** |  | 1.00 | 0.95 | 1.06 | 0.98 | 0.82 | 1.13 | 1.11 | 1.07 | 1.15 | 0.90 | 0.81 | 1.00 | 1.09 | 1.01 | 1.17 | 1.03 | 0.98 | 1.09 | 0.89 | 0.86 | 0.92 |
| **Boys** |  | 0.79 | 0.50 | 1.08 | 1.18 | 0.46 | 1.91 | 0.71 | 0.51 | 0.91 | 0.45 | 0.01 | 0.90 | 0.05 | -0.42 | 0.53 | 0.59 | 0.32 | 0.85 | 0.55 | 0.38 | 0.73 |
| **Girls** |  | 0.00 | . | . | 0.00 | . | . | 0.00 | . | . | 0.00 | . | . | 0.00 | . | . | 0.00 | . | . | 0.00 | . | . |
| **Age*sex** | **Boys** | -0.28 | -0.41 | -0.15 | -0.41 | -0.69 | -0.13 | -0.22 | -0.32 | -0.13 | -0.18 | -0.37 | 0.01 | -0.01 | -0.22 | 0.21 | -0.23 | -0.35 | -0.11 | -0.21 | -0.29 | -0.14 |
|  | **Girls** | 0.00 | . | . | 0.00 | . | . | 0.00 | . | . | 0.00 | . | . | 0.00 | . | . | 0.00 | . | . | 0.00 | . | . |
| **(Age^2^)*sex** | **Boys** | 0.02 | 0.01 | 0.03 | 0.03 | 0.01 | 0.05 | 0.01 | 0.00 | 0.02 | 0.01 | 0.00 | 0.03 | 0.00 | -0.01 | 0.02 | 0.02 | 0.01 | 0.03 | 0.01 | 0.01 | 0.02 |
|  | **Girls** | 0.00 | . | . | 0.00 | . | . | 0.00 | . | . | 0.00 | . | . | 0.00 | . | . | 0.00 | . | . | 0.00 | . | . |
| **log(Age)*sex** | **Boys** | 0.11 | 0.04 | 0.18 | 0.19 | 0.03 | 0.36 | 0.11 | 0.06 | 0.16 | 0.02 | -0.09 | 0.13 | -0.02 | -0.13 | 0.09 | 0.06 | -0.01 | 0.13 | 0.14 | 0.10 | 0.19 |
|  | **Girls** | 0.00 | . | . | 0.00 | . | . | 0.00 | . | . | 0.00 | . | . | 0.00 | . | . | 0.00 | . | . | 0.00 | . | . |
| **Pre-term birth: yes** |  | 0.22 | -0.36 | 0.80 | -1.40 | -3.37 | 0.57 | 0.10 | -0.27 | 0.47 | 0.61 | -0.29 | 1.52 | -0.19 | -1.21 | 0.84 | 0.75 | 0.25 | 1.25 | 0.13 | -0.29 | 0.54 |
| **Pre-term birth: no** |  | 0 |  |  | 0 |  |  | 0 |  |  | 0 |  |  |  |  |  | 0 |  |  | 0 |  |  |
| **Age*pre-term birth** | **Yes** | -0.28 | -0.55 | -0.01 | 0.27 | -0.51 | 1.06 | -0.23 | -0.41 | -0.06 | -0.46 | -0.87 | -0.04 | -0.29 | -0.78 | 0.20 | -0.55 | -0.79 | -0.31 | -0.28 | -0.47 | -0.10 |
|  | **No** | 0 |  |  | 0 |  |  | 0 |  |  | 0 |  |  |  |  |  | 0 |  |  | 0 |  |  |
| **(Age^2^)*pre-term** | **Yes** | 0.01 | -0.01 | 0.04 | -0.01 | -0.07 | 0.05 | 0.02 | 0.00 | 0.03 | 0.03 | 0.00 | 0.07 | 0.03 | -0.01 | 0.07 | 0.04 | 0.01 | 0.06 | 0.02 | 0.00 | 0.04 |
|  | **No** | 0 |  |  | 0 |  |  | 0 |  |  | 0 |  |  |  |  |  | 0 |  |  | 0 |  |  |
| **log(Age)*pre-term birth** | **Yes** | 0.41 | 0.26 | 0.57 | -0.15 | -0.59 | 0.30 | 0.24 | 0.14 | 0.35 | 0.32 | 0.07 | 0.58 | 0.18 | -0.06 | 0.42 | 0.61 | 0.47 | 0.76 | 0.50 | 0.38 | 0.62 |
|  | **No** | 0 |  |  | 0 |  |  | 0 |  |  | 0 |  |  |  |  |  | 0 |  |  | 0 |  |  |
| **Reported weight: yes** |  | 0.38 | 0.21 | 0.54 | 0.29 | -0.09 | 0.66 | -0.02 | -0.25 | 0.20 | -0.66 | -0.90 | -0.42 | 0.03 | -0.15 | 0.21 | 0.07 | -0.10 | 0.24 | -0.94 | -1.41 | -0.47 |
| **Reported weight: no** |  | 0 |  |  | 0 |  |  | 0 |  |  | 0 |  |  |  |  |  | 0 |  |  | 0 |  |  |

**Table C:** Results of the basic adjusted model for BMI growth: effect estimates and 99% confidence intervals stratified by country; model adjusted for sex, pre-term birth and respective age interactions

LCI: lower confidence interval

UCI: upper confidence interval


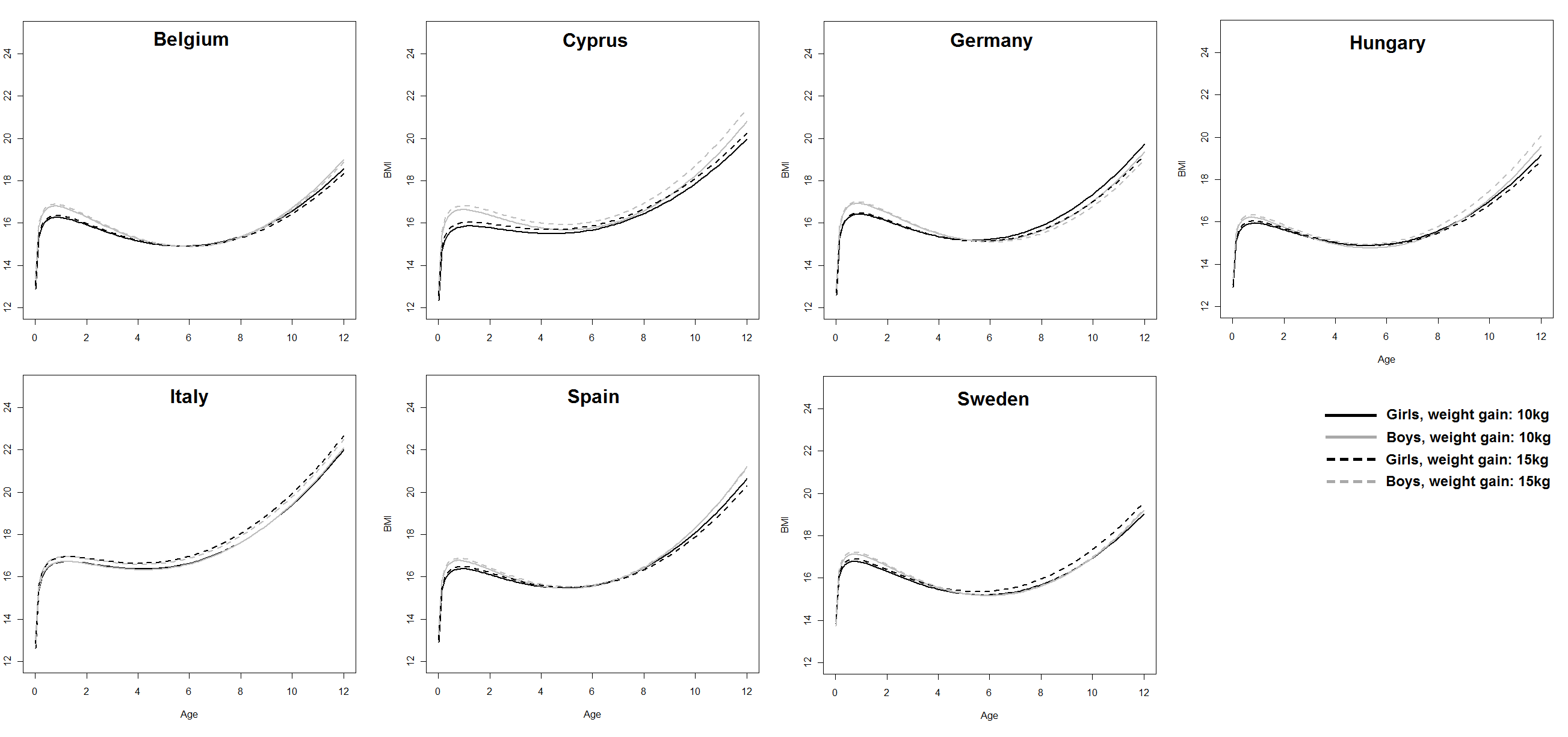


**Fig A:** Predicted BMI growth trajectories of children having a mother with a gestational weight gain of 10 kg (solid) vs. 15 kg (dashed) by sex (girls: black; boys: grey) and country; all other covariates were set to a constant value (continuous covariates were set to 0 (i.e. to the value used for centring), categorical variables were set to the reference category)


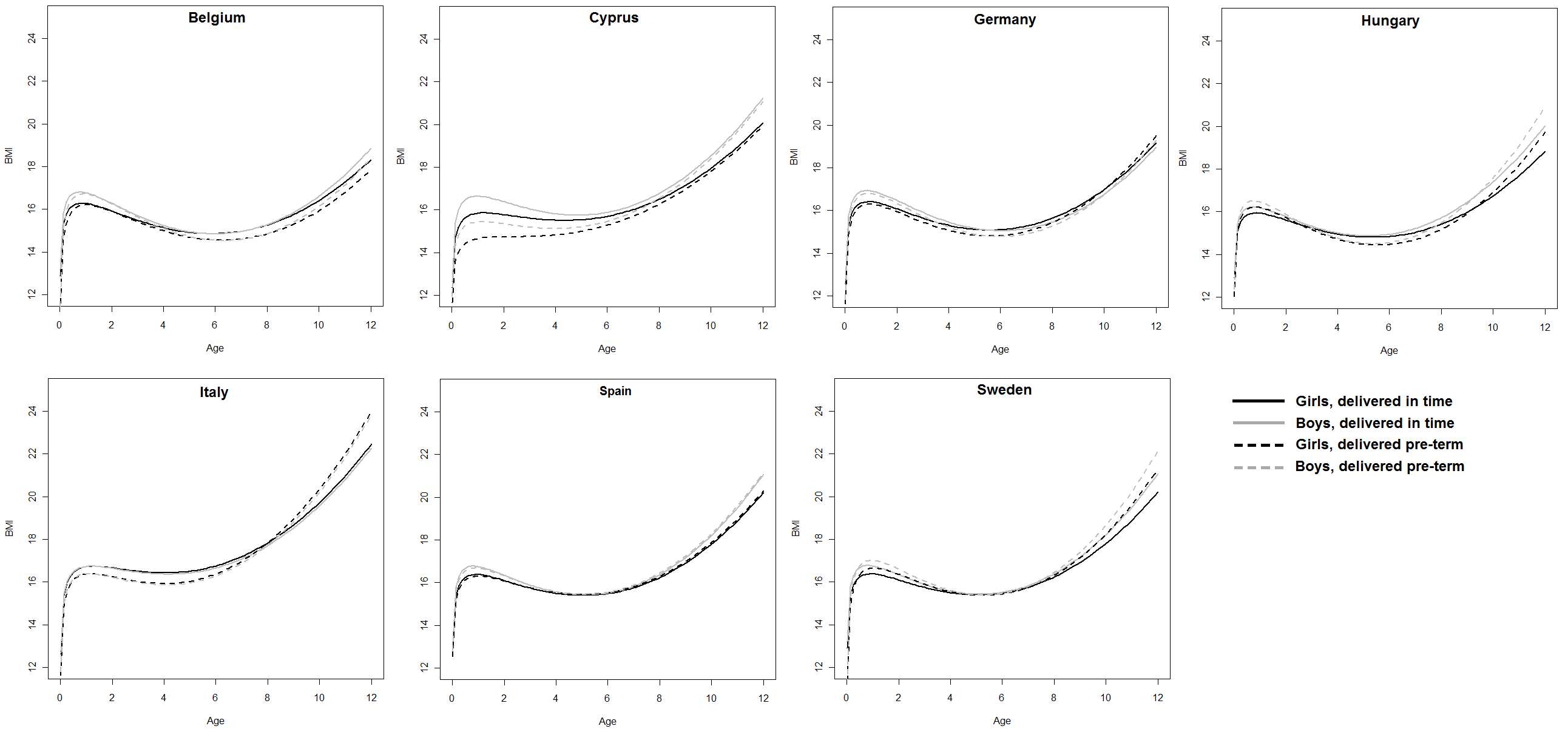


Fig B: Predicted BMI growth trajectories of children delivery at term (solid) vs. pre-term (dashed) by sex (girls: black; boys: grey) and country; all other covariates were set to a constant value (continuous covariates were set to 0 (i.e. to the value used for centring), categorical variables were set to the reference category)


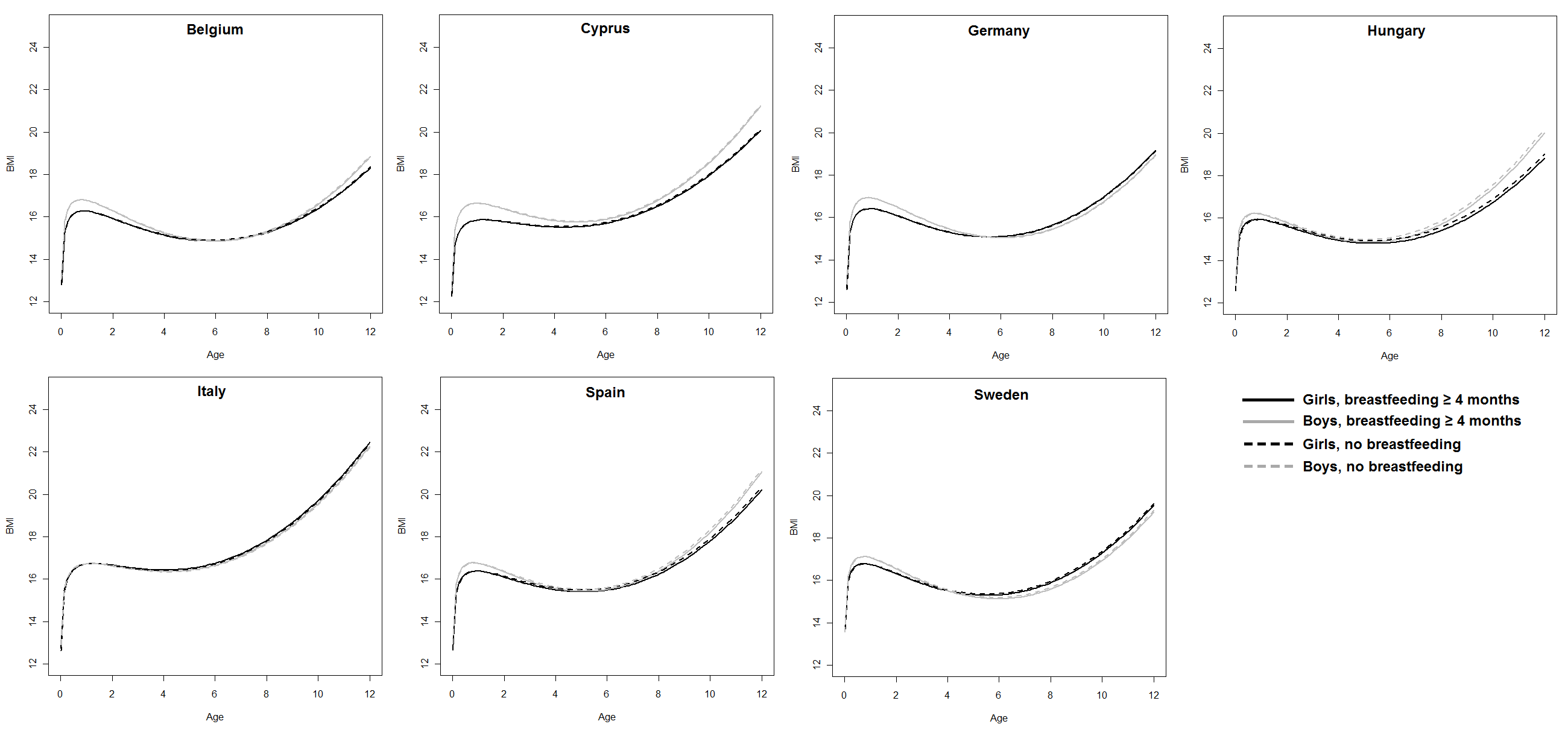


Fig C: Predicted BMI growth trajectories of children being breast fed for ≥ 4 months (solid) vs. not being breast fed (dashed) by sex (girls: black; boys: grey) and country; all other covariates were set to a constant value (continuous covariates were set to 0 (i.e. to the value used for centring), categorical variables were set to the reference category)


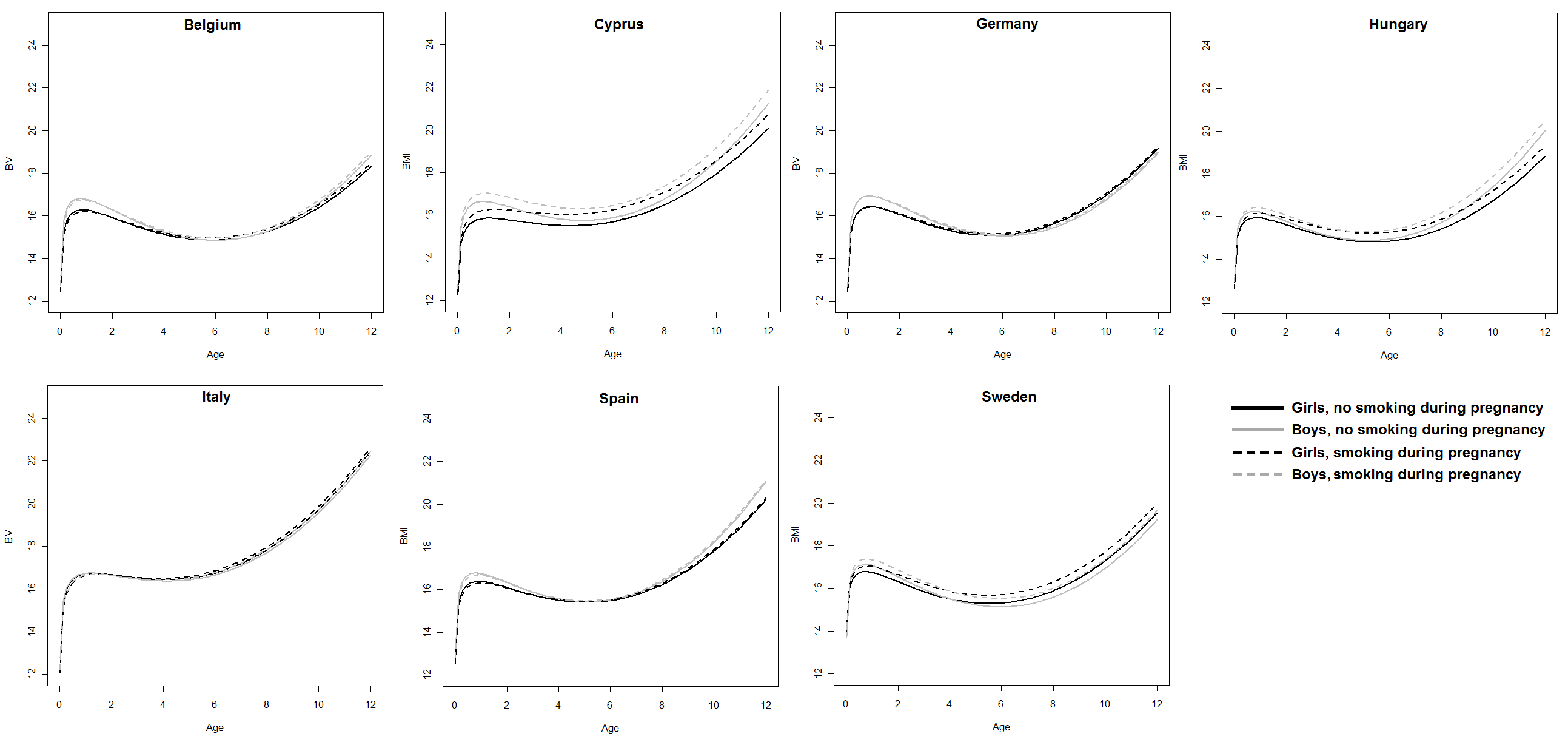


Fig D: Predicted BMI growth trajectories of children whose mothers did not smoke (solid) vs. smoked during pregnancy (dashed) by sex (girls: black; boys: grey) and country; all other covariates were set to a constant value (continuous covariates were set to 0 (i.e. to the value used for centring), categorical variables were set to the reference category)
